# Supplementary material for: Biomimetic 3D Models for Investigating the Role of Monocytes and Macrophages in Atherosclerosis
Source: Bioengineering (Basel). 2020 Sep 16;7(3):113. doi: 10.3390/bioengineering7030113 (PMC7552756; doi:10.3390/bioengineering7030113)
Supplement: Supplementary file 1 [file bioengineering-07-00113-s001.pdf]

bioengineering

MDPI

Supplementary Materials

Table 1. Primer list.

Gene		Primer Sequences
**LOX1**	Forward:<br>Reverse:	ACGGACAGACAGACAGACAG<br>GGCCACACATCCCATGATTC
CD36	Forward:<br>Reverse:	TGGTACAGATGCAGCCTCAT<br>AGGCCTTGGATGGAAGAACA
CD68	Forward:<br>Reverse:	CATGGCGGTGGAGTACAATG<br>GCAGGAGAAACTTTGCCCAA
MHCII	Forward:<br>Reverse:	TCCTGGTCCAACTTCTGTCC<br>CCCAACCTCATCCGATCTGA
ACTB	Forward:<br>Reverse:	CATCCGCAAAGACCTGTACG<br>CCTGCTTGCTGATCCACATC

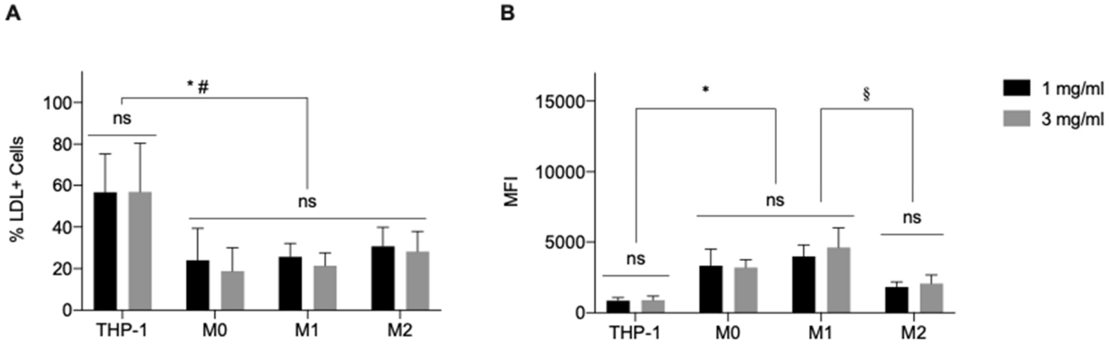

Figure 1. Fluorescence assisted cell sorting assessment of LDL uptake by monocytes and macrophages cultured in 3D collagen hydrogels. **(A)**The number of (oxLDL+) cells for THP-1 monocytes and THP-1-derived macrophages. (B) The amount of LDL in the cells, as quantified through mean fluorescence intensity (MFI), is also shown. A sample size of $n = 5$ was performed. Significance is represented as * $p < 0.05$ between THP-1 *vs.* M0 and M1, § $p < 0.05$ between M1 and M2, and # $p < 0.1$ between 1 mg/ml and 3 mg/ml THP-1, and M2 at the same matrix conditions.
